# Establishment of Analytical Model for CFRP Cutting Force Considering the Radius of the Edge Circle

**DOI:** 10.3390/ma15062127

**Published:** 2022-03-14

**Authors:** Haifeng Ning, Hualin Zheng, Guixin Wang

**Affiliations:** School of Mechatronic Engineering, Southwest Petroleum University, Chengdu 610000, China; ninghf1978@163.com (H.N.); wgx2282577949@163.com (G.W.)

**Keywords:** CFRP, cutting force, finite element, fiber cutting angle, analytical model

## Abstract

Carbon fiber-reinforced composite material (CFRP) has been widely applied in the aerospace industry, which places demanding requirements on the accuracy and quality of its processing. However, there remains a lack of clarity on the microscopic material removal process of CFRP, despite substantial relevant research. This paper aims to reveal the mechanism of material removal in the CFRP cutting process at different fiber cutting angles and to establish an analytical model for CFRP cutting force by considering the radius of the edge circle. Furthermore, the CFRP cutting force analytical model was established by considering the radius of the edge circle on the basis of the CFRP representative volume unit (RVE). According to the model, the cutting process was divided into three regions, the cutting slip zone, fiber fracture zone, and spring back zone, with consideration given to the effect of residual fibers on the cutter teeth. The CFRP cutting finite element model was defined using the software Abaqus, while the chip removal and single-fiber deformation processes were analyzed using the finite element model. As indicated by the experimental results, the analytical model is reliable and capable of providing cutting force values within a 15% deviation.

## 1. Instruction

Currently, carbon fiber-reinforced composite material (CFRP) is widely applied in the aerospace industry due to its high specific strength and excellent performance in corrosion resistance [1,2]. In order to meet the assembly-related requirements, it is often necessary to process CFRP, such as turning and milling, as the aerospace industry has high requirements for the quality of CFRP processing [3,4]. However, CFRP is a typical anisotropic material. The removal mechanism of the material is different from that of metal, and it is largely affected by the cutting angle of the fiber [5]. The strength of carbon fiber is much greater than that of epoxy resin matrix, and various defects such as tearing burrs are often caused during processing. As one of the most important influencing factors for the quality of CFRP processing, cutting force also determines the wear rate of the tool [6,7]. Therefore, it is of great significance to analyze the mechanism of CFRP material removal and accurately predict the cutting force during CFRP processing for better control of CFRP processing damage and tool wear.

Scholars at home and abroad have conducted substantial research on the CFRP cutting process using finite element and cutting force analytical models. Calzada et al. [8] established a microscopic simulation model by finite element software, in which the interface phase was simulated by continuous elements. The model was used to simulate the failure mode of fibers when chips were formed in the cutting process, and the accuracy of the model was verified through experiments. Dandekar et al. [9] established a two-dimensional cutting model with an interface phase of zero thickness in order to simulate the failure mode of the fiber matrix under different fiber direction angles during the cutting process. The simulation results showed that a larger fiber direction angle led to more debonded fiber and resin matrix. Liu et al. [10] established a micro-orthogonal cutting model in order to more realistically simulate the material removal process, fiber matrix failure, and interface cracking. The simulation results showed that, when the fiber direction angle was 0° and 135°, the fiber failed due to bending and fracture. When the fiber direction angle was 45° and 90°, the fiber matrix failed due to the squeezing action of the cutter. Wang et al. [11] conducted a two-dimensional microscopic model CFRP cutting simulation to explore the material removal mechanism during the cutting process, and they found that the material removal was mainly related to fiber fracture and interface cracking. Chen et al. [12] established macroscopic and microscopic CFRP cutting models and found that the fiber direction was the main factor affecting the processing quality. The surface quality was poor when the fiber direction angle was 0° and 135°, while the surface quality under the directions of 90°and 45° was better. Puw et al. [13] proposed a bending failure model of cutting chips perpendicular to the fiber axis based on beam theory, linear elastic fracture mechanics, and composite mechanics, and they established the relationship between cutting force and chip length. Zhang et al. [14] divided the cutting zone into three parts: chip zone, extrusion zone, and fiber rebound zone, and they established a theoretical model of composite material cutting force with a fiber cutting angle of 0°–90° using contact mechanics. Qi et al. [15] established an analytical model of 0° ≤ *θ* ≤ 90° carbon fiber cutting force based on RVE using the principle of minimum potential energy. Xiao et al. [16] also established an analytical model of CFRP considering the interface phase based on RVE and used this model to analyze the processing damage of CFRP under different fiber cutting angles. Voss et al. [17] first studied the real wear of the tool under different fiber cutting angles and then established a theoretical model of CFRP cutting force considering tool wear. This model was suitable for fiber cutting angles of 0°–180°.

To sum up, most of the studies on the CFRP cutting process of finite elements were aimed at analyzing the chip formation mechanism and cutting quality, with less attention paid to the mechanical behavior of a single fiber in the cutting process. Although the theoretical analysis model of CFRP cutting force has been improved significantly to date, the theoretical analysis model is still not linked to the single-fiber fracture process, and there are still many factors that are ignored. Therefore, this paper aimed to establish a CFRP micro-cutting finite element model, analyze the CFRP material removal process and single-fiber mechanical behavior, and establish an analytical model of CFRP cutting force (0° < *θ* ≤ γ + 90°) by considering the edge radius. Lastly, the accuracy of the model is verified through experiment.

## 2. CFRP Finite Element Micro-Cutting Model

**Figure 1:**
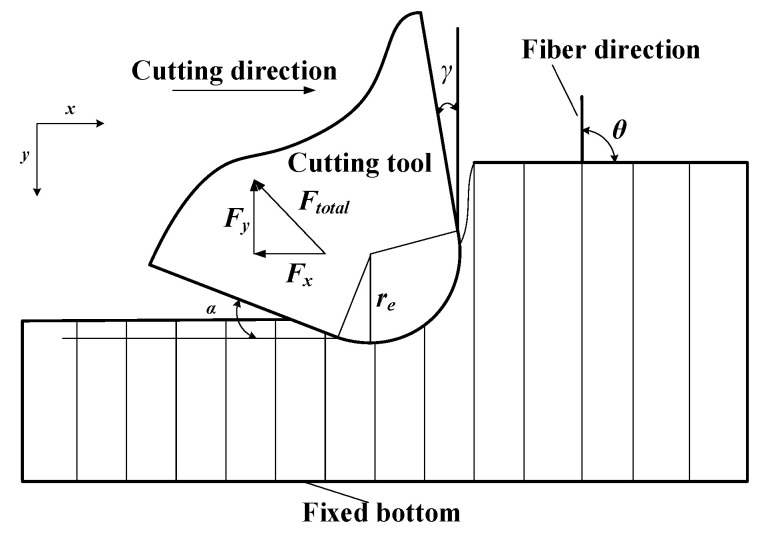
The schematic of fiber cutting angle.

The objective of this study was to develop a model for cutting forces involved in CFRP machining, considering the range of fiber cutting angle 0° < *θ* ≤ γ + 90°, as shown in Figure 1, where *r_e_* is the radius of the edge circle, γ is the tool rake angle, and α is the tool clearance angle. The fiber cutting angle *θ* is the angle between the cutting direction and the fiber direction. During CFRP cutting, the total cutting force *F*_total_ can be divided into horizontal force *F*_x_ and vertical force *F*_y_ [18].

### 2.1. Geometric Model and Mesh

**Figure 2:**
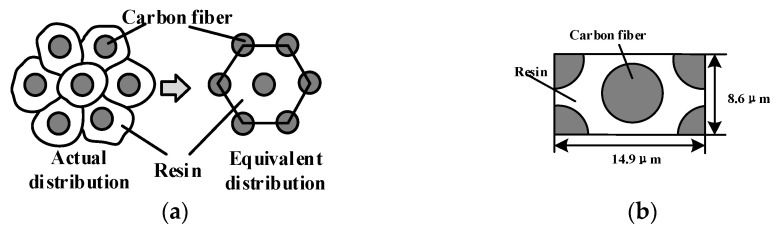
The schematics illustration of carbon fiber distribution processing: (**a**) CFRP microscopic arrangement; (**b**) definition of the equivalent section of the element body, preserving the geometry and the volume fraction of fiber to matrix.

In order to study the removal process of fiber and matrix in the processing of CFRP and establish an analytical model of CFRP cutting force on this basis, the finite element software Abaqus (Dassault Systemes Simulia Corp, Paris, France) was used to establish a CFRP cutting model. The finite element simulation model takes *θ* = 90° as an example to analyze the CFRP cutting process. Carbon fiber filaments have a certain randomness in the cross-section. In order to better simulate the fiber cutting process, the carbon fiber distribution in the yarn was set equivalent to a hexagonal arrangement in the matrix [19]. The relevant parameters of CFRP are presented in Section 5. The unit calculation body can characterize the distribution characteristics of carbon fiber in the matrix. In order to facilitate the analysis, the unit calculation body method is used in this paper, as shown in Figure 2 [16]. According to the volume fraction, the length and width of the unit calculation body can be calculated as 14.9 μm and 8.6 μm, respectively.

**Figure 3:**
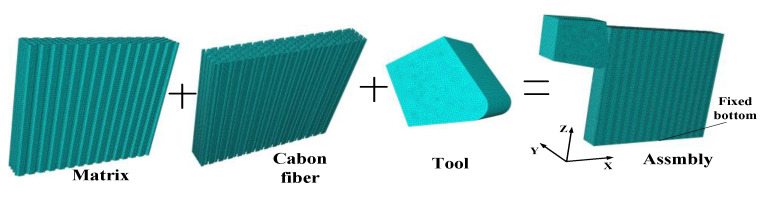
Finite element mesh.

In the CFRP micro-cutting simulation process, if the number of unit calculation bodies is too large, it will affect the calculation speed, and, if the number of units is too small, the interaction between the fibers in the simulation cannot be simulated, which will affect the observation of the CFRP removal mechanism. According to the research results of Xiao et al., the thickness direction of the model adopts a four-unit calculation body [16]. Therefore, the thickness of the CFRP model was 34.4 μm. The tool edge radius *r_e_* was 15 μm, the rake angle *γ* was 10°, and the clearance angle α was 15°. The cutter, fiber, and matrix all used solid elements. The explicit dynamic model was used to calculate this model. The element type selected to mesh all the bodies was an eight-node reduced integral element (C3D8R), which can avoid the reduction in calculation accuracy caused by excessive distortion of the element. Considering the calculation accuracy and efficiency at the same time, this is more suitable when the grid size is 1.5–2.5 μm [19,20,21]. To make the finite element converge, the selection of tool and material mesh size should match. The fiber element size was 1.5 μm, and the matrix and cutter element size was 2 μm. After the fiber matrix and the tool were drawn as meshes, they were assembled in the assembly module. Too much distance between the tool and the CFRP assembly would increase the amount of calculation; thus, the initial position of the tool was 2 μm away from the CFRP, as shown in Figure 3.

### 2.2. Material Parameters and Boundary Conditions

The CFRP cutting simulation mainly set the material properties of carbon fiber, epoxy resin, and the tool, as well as the bonding between the fiber and the matrix, etc. Carbon fibers are defined as a transversely isotropic material with elastic deformation and no plastic deformation. The failure criterion of carbon fiber adopted the maximum stress criterion, specifically expressed as follows [19]:

(1) $$\mathrm{Tensile}\;\mathrm{failure}\;\mathrm{in}\;\mathrm{longitudinal}\;\mathrm{direction}\;\;\;\;(\sigma_{11}\geq 0)\;\;\;\;{\left(\frac{\sigma_{11}}{X_{T}}\right)}^{2}\geq 1,\;\mathrm{Compressive}\;\mathrm{failure}\;\mathrm{in}\;\mathrm{longitudinal}\;\mathrm{direction}\;\;\;\;(\sigma_{11}<0)\;\;\;\;{\left(\frac{\sigma_{11}}{X_{C}}\right)}^{2}\geq 1,\;\mathrm{Tensile}\;\mathrm{failure}\;\mathrm{in}\;\mathrm{transverse}\;\mathrm{direction}\;\;\;\;(\sigma_{22}\geq 0\;or\;\sigma_{33}\geq 0)\;\;\;\;{\left(\frac{\sigma_{22}}{Y_{T}}\right)}^{2}\geq 1,{\left(\frac{\sigma_{22}}{Y_{T}}\right)}^{2}\geq 1,\;\mathrm{Compressive}\;\mathrm{failure}\;\mathrm{in}\;\mathrm{transverse}\;\mathrm{direction}\;\;\;\;(\sigma_{22}<0\;or\;\sigma_{33}<0)\;\;\;\;{\left(\frac{\sigma_{22}}{Y_{C}}\right)}^{2}\geq 1,{\left(\frac{\sigma_{33}}{Y_{C}}\right)}^{2}\geq 1,$$

where *σ_ii_* is the stress tensor component of the fiber, the 11-principal direction refers to the fiber direction, while the 22- and 33-principal directions refer to the transverse (isotropic) plane direction, *X_T_* and *X_C_* are the tensile and compressive strengths in the longitudinal direction, and *Y_T_* and *Y_C_* are the tensile and compressive strength in the transverse direction.

**Figure 4:**
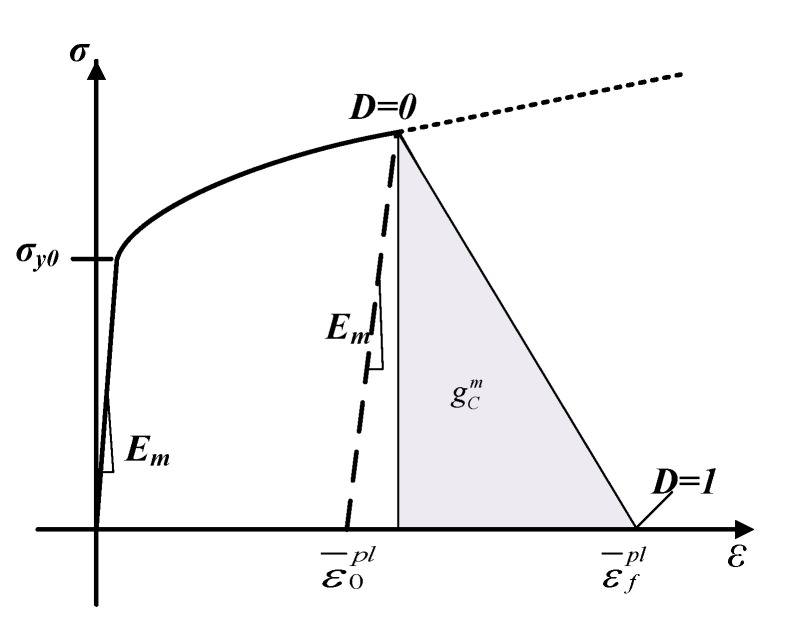
Stress–strain constitutive relationship of resin matrix.

The epoxy resin matrix is defined as an isotropic material with elastoplastic deformation. The stress–strain constitutive relationship of the resin matrix is shown in Figure 4. The solid line and the dashed line respectively indicate the stress–strain relationship of the material when material damage is considered and not considered. The elasticity of the resin matrix is defined by Poisson’s ratio and the elastic modulus. When the stress reaches the yield stress *σ_y_*_0_, the material enters the plastic stage, and the Johnson–Cook constitutive model is used to describe the material constitutive relationship in the plastic stage. When the equivalent plastic strain reaches ${\bar{\varepsilon }}_{0}^{pl}$, the material begins to be damaged. The expression of the degraded stiffness matrix is as follows:

(2) $$E_{m}^{\prime }=(1-d_{m})E_{m},$$

where $E_{m}$ is the elastic modulus of the matrix, $E_{m}^{\prime }$ is the degraded stiffness matrix, and *d_m_* is the damage variable. The initial value of the damage variable is 0; if *d_m_* = 1, the element has completely failed and the stiffness is completely degraded. When the equivalent strain reaches ${\bar{\varepsilon }}_{f}^{pl}$, the material has failed completely. At this point, the fracture energy density $g_{C}^{m}$ reaches the fracture energy $G_{C}^{m}$.

**Figure 5:**
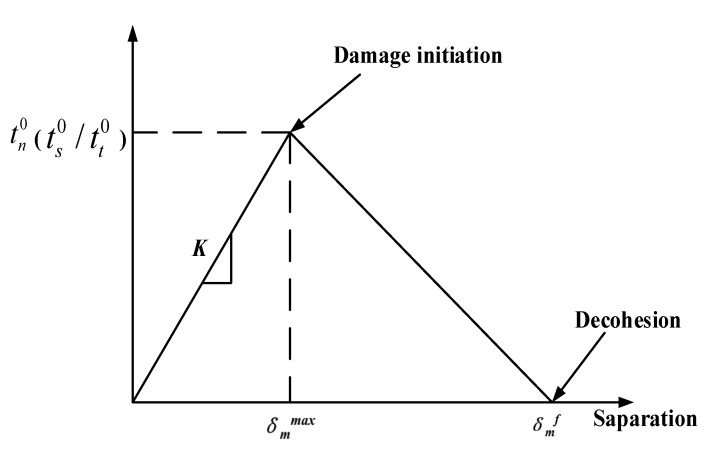
The response of the interface phase linear elastic separation under normal loading.

Considering that the model uses a grid made of fiber and matrix parts (see Figure 3), an interface must be defined, connecting the discrete phases to transfer stress to each other. The interface transmits stresses as long as there is a contact of fiber and matrix. However, the interface can also fail due to excessive cutting force during the machining process. This paper chose the surface-based cohesive behavior method. This method uses cohesive behavior as the contact attribute, which is beneficial to avoid excessive deformation of cohesive units and improve calculation efficiency. Meanwhile, this method has the advantages of easy definition and is often used for the simulation of cohesive mutual behavior. Figure 5 shows the traction stress (t) response of the interface phase linear elastic separation under normal loading. $\delta_{m}^{\max}$ is the maximum value of the effective separation displacement, and $\delta_{m}^{f}$ is the separation displacement at complete failure. Under the action of a cutting force, the nondamaged stage is a linear elastic response. The interface begins to deteriorate if the stress reaches the elastic limit (*t*^0^), and, from that point, the stiffness is degraded, which can be interpreted as the starting and growing of a crack at the interface. The fibers are gradually separated from the matrix, and the interface stiffness is degraded. When the interface cracks to a certain extent, the fiber and the matrix are completely separated [22,23].

The elastic constitutive equation of the interface phase is

(3) $$t=\left\{\begin{array}{c} t_{n}\\ t_{s}\\ t_{t} \end{array}\right\}=\left[\begin{array}{ccc} K_{nn} & 0 & 0\\ 0 & K_{SS} & 0\\ 0 & 0 & K_{tt} \end{array}\right]\left\{\begin{array}{c} \delta_{n}\\ \delta_{s}\\ \delta_{t} \end{array}\right\}=K\delta ,$$

where *t* is the nominal traction stress, *t_n_* is the normal stress at the interface, *t_s_* and *t_t_* are shear stresses at the interface, and $\delta_{n}$, $\delta_{s}$, $\delta_{t}$ are the interface separation distances corresponding to the three stresses.

In this paper, the maximum stress criterion was selected to simulate the interface damage. When the maximum stress ratio reaches 1, damage begins to occur. Its expression is as follows:

(4) $$\max\left\{\frac{t_{n}}{t_{n}^{0}},\frac{t_{s}}{t_{s}^{0}},\frac{t_{t}}{t_{t}^{0}}\right\}=1.$$

After the damage starts, the Benzeggagh–Kenane (BK) fracture criterion was used as the cohesive failure criterion, expressed as follows [22]:

(5) $$G_{1}^{C}+(G_{2}^{C}-G_{1}^{C}){\left\{\frac{G_{S}}{G_{T}}\right\}}^{\eta_{\alpha }}=G^{C},$$

where *G*_1_, *G*_2_, and *G*_3_ are pure interface energy ($G_{S}=G_{2}+G_{3}$ and $G_{T}=G_{1}+G_{2}+G_{3}$), and *η_α_* is a mixed-mode parameter.

CFRP material has the characteristics of high hardness and strong wear resistance; hence, cemented carbide tools were selected. The elastic modulus of the cutting tool is much greater than that of carbon fiber and epoxy resin materials. Because of that characteristic, and in order to reduce the calculation demands, the tool was set as a rigid body. The wear of the tool was not considered in the simulation. Related parameters of the CFRP and tool remained the same as described in Section 5.

In the coordinate system shown in Figure 3, the horizontal right direction is the positive direction of the *x*-axis, the thickness direction of CFRP is the *y*-axis, and the vertical upward direction is the positive direction of the *z*-axis. In the experiment, the bottom edge of the workpiece was generally clamped; hence, the bottom surface of the CFRP in the finite element was completely fixed and restrained. The speed *v* in the *x*-direction was set on the tool. The contact of the parts was defined considering the ‘hard contact’ condition for normal behavior and the ‘penalty function’ for tangential behavior. The values of the tool–fiber and fiber–fiber coefficients of friction were both 0.2. The fiber–matrix contact could be ignored, as the matrix material was easily removed while cutting. The tool–matrix friction coefficient was set to 0.3 [16].

## 3. Analysis of Finite Element Simulation Results

**Figure 6:**
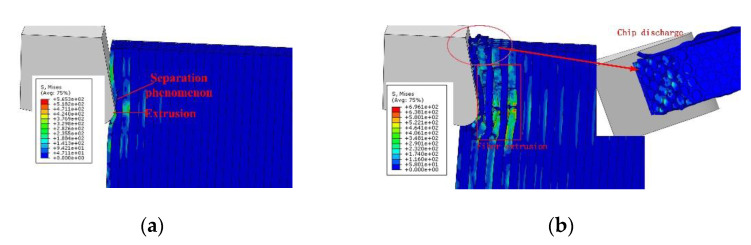
Stress (von Mises) values of the CFRP workpiece calculated with the FE model, with fiber angle *θ* = 90°, showing the initial extrusion (**a**) and the chip formation (**b**). The workpiece was fixed at the bottom surface.

In Figure 6, the stress values at the CFRP calculated with the FE model are shown with fiber angle *θ* = 90°, showing the initial extrusion (a) and the chip formation (b). The material properties and tool parameters are detailed in Section 5, corresponding to those used in the test case. It can be seen from Figure 6a that the CFRP first contacted the tip of the tool during the cutting process and was squeezed. The carbon fibers were supported by the back fibers. As the matrix and interface strength values were lower than for the fiber, their failure happened first, as described in Section 1. Those broken mesh elements were removed from the mesh for the next iterations. Fibers also broke when the element stress reached the ultimate stress value, and mesh elements were removed. Broken carbon fibers were discharged along the rake surface and continued to squeeze the back fibers. During this process, the squeezing effect between the fibers was strong, resulting in obvious separation between the fibers and the matrix, as shown in Figure 6b.The broken carbon fiber was broken again under the action of the tool tip, and the broken point of some fibers was below the cutting plane.

**Figure 7:**
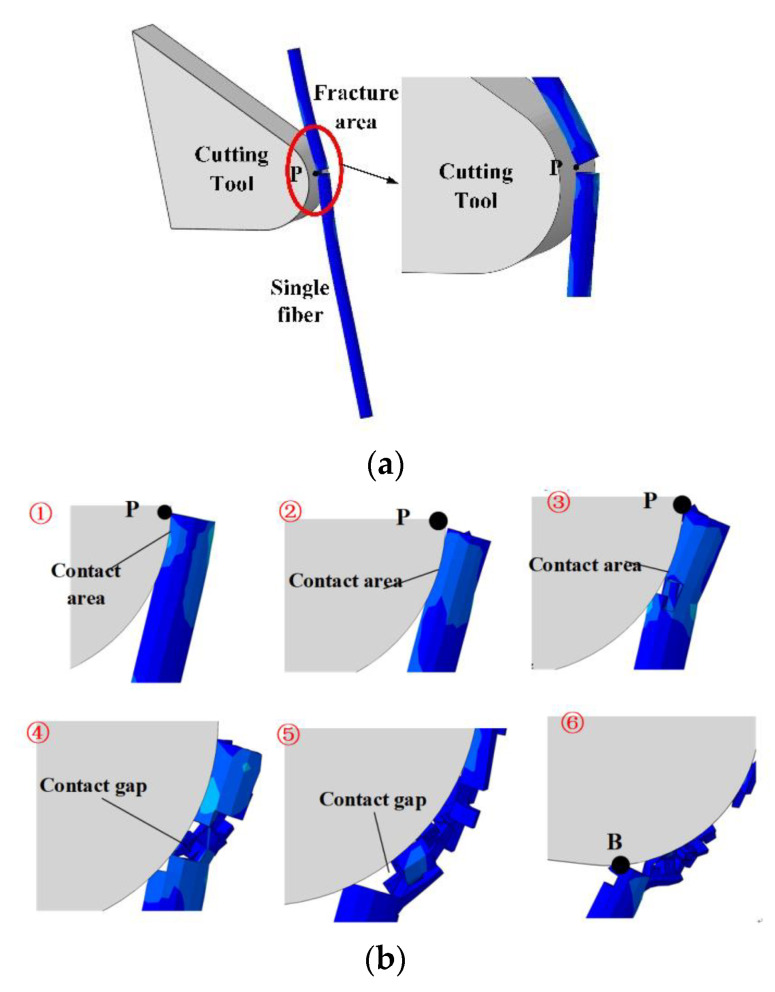
Single-fiber removal process: (**a**) primary fracture of fiber; (**b**) residual fiber removal.

Figure 7 shows the breaking sequence of a single fiber provided by the FE model. The cutter tooth and the fiber first came into contact at point P, and the fiber broke at that point (Figure 7a). The involved fiber mesh elements were removed. The material gap is visible. The residual fiber mesh elements interacted with the cutter teeth near point P. With the movement of the tool, the contact zone between the cutter teeth and the carbon fiber gradually moved down along the teeth surface (Figure 7b). It should be noted that, in this process, the residual fiber was not in contact with the cutter teeth at the free end. The tool movement involved the further deformation of the fiber, and more fiber elements could break and be removed. This process was not stable, and the contact between the fiber and the cutter teeth was discontinuous, including gaps. The process went on until the residual fiber passed through the lowest point B of the cutter.

## 4. CFRP Cutting Force Analytical Model

**Figure 8:**
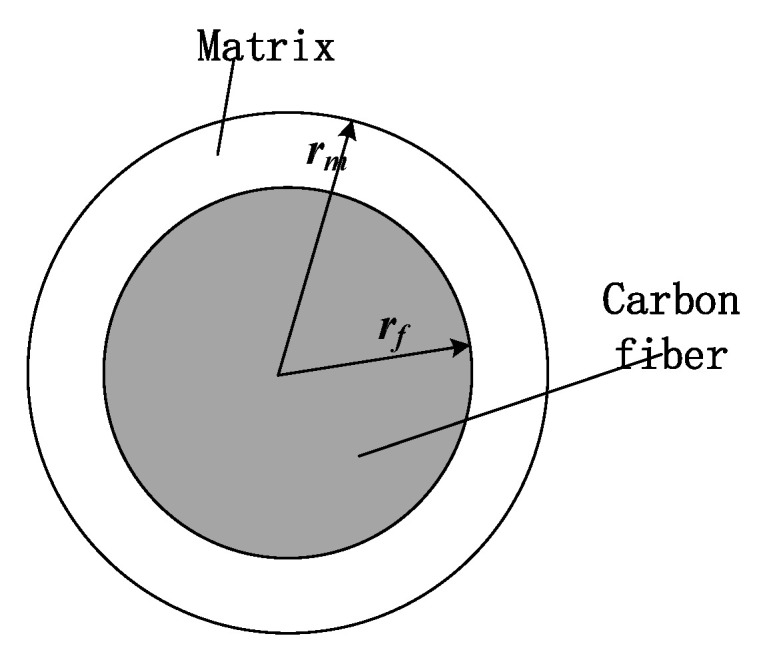
The schematic illustration of RVE cross-section.

On the basis of previous studies, a semi-infinite length representative volume element (RVE) model was used to establish a CFRP cutting force analytical model considering the radius of the edge circle. The schematic diagram of the RVE cross-section is shown in Figure 8, where *r_f_* is the radius of the carbon fiber, and *r_m_* is the radius of the RVE, obtained according to the equivalent volume method $r_{m}=\frac{r_{f}}{\sqrt{V_{f}}}$ [15].

**Figure 9:**
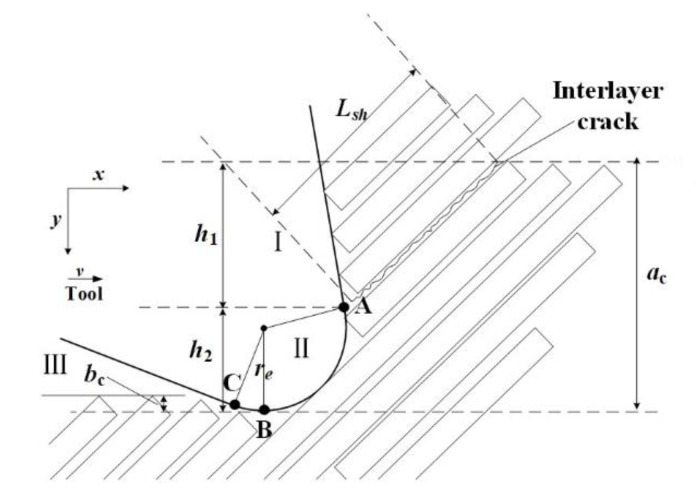
Schematic illustration of the CFRP cutting process. The tip of the tool is surrounded by fibers, some already broken. The zones of slip (I), fracture (II), and rebound (III) are clearly visible. The depths *a_c_*, *h*_1_, and *h*_2_ are defined in the drawing.

Drawing on the theory of metal cutting deformation [21], the cutting process can be divided into three zones: cutting slip zone (zone I), fiber resin fracture zone (zone II), and rebound zone (zone III). In the cutting process, the tip of the tooth first contacts the RVE, as shown in Figure 9. After the RVE is cut (zone II), one part flows out along the rake face (zone I), and another part after cutting rebounds to have an effect on the flank face (zone III).

Since the analysis depends directly on the fiber cutting angle (*θ*), it was applied for the general range 0°< *θ* ≤ *γ* + 90°, where *γ* is the tool rake angle, as shown in Figure 1 and Figure 9. Two cases, *θ* = 45° and 90°, were selected as an example for this study. Indeed, the two cases are directly implemented as test cases for comparison in Section 5.

The cutting depth is *a_c_* (see Figure 9), and the cutting depths *h*_1_ and *h*_2_ of zone I and II are calculated according to the following geometric relationships:

(6) $$h_{1}=a_{c}-(1+\sin\gamma )r_{e},$$

(7) $$h_{2}=(1+\sin\gamma )r_{e}.$$

### 4.1. Cutting Slip Zone (Zone I)

It is assumed that, after the fibers in zone II are cut, the fibers at the upper end immediately contact the rake face. Under the action of the rake face, the carbon fibers are separated from the matrix, and then discharged in the form of chips, no longer in contact with the teeth. According to the research results of Zhang et al. [14], the shear force component of zone I is calculated as

(8) $$P_{\mathrm{sh}}=\tau_{c}L_{sh}b=\tau_{c}\frac{h_{1}}{\sin\theta }b,$$

where *L_sh_* is the single-fiber peeling length (see Figure 9), $\tau_{c}$ is the shear strength of the matrix, and *b* is the thickness of the CFRP laminate.

Considering the friction coefficient (*μ*) between CFRP and the rake face, the cutting force component of zone I can be obtained.

(9) $$F_{1\_x}=\tau_{c}\frac{h_{1}}{\sin\theta }b(\cos\theta +\mu \cos(\theta -\gamma )\sin\gamma ),\;F_{1\_y}=\tau_{c}\frac{h_{1}}{\sin\theta }b(-\sin\theta +\mu \cos(\theta -\gamma )\cos\gamma ),$$

where *h*_1_ is the distance between point A and the upper surface of the workpiece (see Figure 9), and *γ* is rake angle of the tool (see Figure 1).

### 4.2. Fiber Resin Fracture Zone (Zone II)

From the previous finite element analysis process, it can be seen that, after the fiber in the second zone is first fractured, there is still residual CFRP material that contacts the cutter; hence, secondary fracture will occur, and a cutting force will also be generated [17]. Therefore, the fracture process of the fiber in contact with zone II can be divided into the following two steps:

1. Fracture occurs when the fiber is in contact with the tool for the first time,
2. The residual material of the fiber further contacts the tool and may cause secondary fractures.

Step 1: Calculation method of the cutting force at first break.

**Figure 10:**
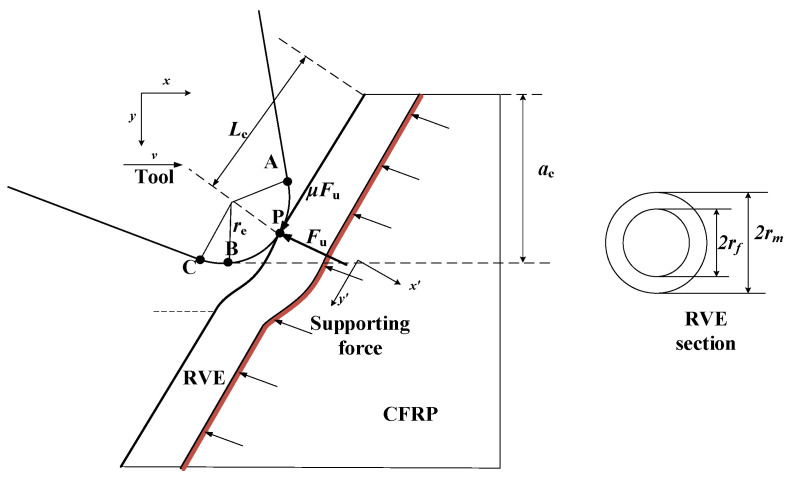
Schematic diagram of the forces acting on the RVE. The cutting tool exerts the force *F_u_* on the RVE, as well as the supporting material on the opposite side.

The theoretical model of CFRP cutting that considers the radius of the edge circle established in this paper is based on extensive previous research [15]. During the modeling process, the influence of the fiber in the thickness direction, the compression of the matrix, and the work done by the internal force of the fiber are ignored. A schematic diagram of the forces acting on the RVE is shown in Figure 10. During the initial contact and first fracture of the fiber, the RVE is mainly subjected to the force *F_u_* of the cutter teeth and the supporting force of the back CFRP. Considering the strain energy of the fiber and the matrix during the initial fracture of the fiber, the expression of the total energy during the process is as follows [15]:

(10) $$\Pi =U_{f}+U_{m}-W_{Fu}-W_{Pb},$$

where $U_{f}$ is the fiber strain energy, $U_{m}$ is the matrix strain energy, and $W_{Fu}$, $W_{P_{b}}$ are the work done by the external force and the supporting force of the composite material, respectively.

The *x’o’y’* coordinate system was established with the first contact point P as the center, and the *y’* direction as the fiber direction. The fiber can be regarded as a slender beam, with its strain energy expressed as follows [24]:

(11) $$U_{f}=\frac{1}{2}\int_{-L_{c}}^{\infty }E_{f2}I_{f}(\frac{d^{2}\omega }{dy^{2}}{)}^{2}dy\prime ,$$

where $E_{f}{}_{2}$ is the transverse elastic modulus of fiber, $I_{f}$ is the moment of inertia of the section, ω is the deflection, and *L_c_* is the length from point P to the upper surface. *L_c_* can be obtained from the following geometric relationships:

(12) $$L_{c}=\frac{a_{c}-r_{e}(1-\cos\theta )}{\sin\theta }\;\;\;\theta \leq 90{}^{\circ},\;L_{c}=\frac{a_{c}-r_{e}(1+\cos\theta )}{\sin\theta }\;\;\;90{}^{\circ}\;<\;\theta \;\leq \;90{}^{\circ}+\gamma .$$

The work done by the cutter tooth is

(13) $$W_{F_{u}}=F_{u}\omega |{}_{y=0}.$$

The strain energy of the matrix is calculated as follows [15]:

(14) $$U_{m}=\frac{1}{2}\int_{-L_{c}}^{\infty }G_{m}\gamma^{2}A_{m}dy=\frac{1}{2}A_{m}G_{m}{\left(\frac{r_{m}}{r_{f}}\right)}^{2}{\int_{-L_{c}}^{\infty }\left(\frac{d\omega }{dy}\right)}^{2},$$

where *A_m_* is the cross-sectional area of resin ($A_{m}=\frac{\pi r_{f}{}^{2}(1-V_{f})}{V_{f}}$), $r_{m}=\frac{r_{f}}{\sqrt{V_{f}}}$, *G_m_* is the shear modulus of the matrix, and *V_f_* is the fiber volume fraction in CFRP.

The back support force of RVE is related to the deflection of the fiber. According to the Winkler foundation beam theory [15,17,25],

(15) $$dp_{b}=-\frac{E_{b}}{H}\omega (y)2r_{m}dy,$$

where *E_b_* is the equivalent elastic modulus, which can be obtained from the following formula [15]:

(16) $$E_{b}=\frac{E_{f2}E_{m}}{E_{f2}+E_{m}},$$

where *E_m_* is elastic modulus of the matrix.

(17) $$H=2kr_{m},$$

where *k* is a dimensionless number; according to [17], the recommended value of *k* is 3.414.

Therefore, it is possible to obtain

(18) $$W_{p_{b}}=\int_{-L_{c}}^{\infty }\omega dp_{b}=-\frac{E_{b}}{k}\int_{-L_{c}}^{\infty }\omega^{2}dy.$$

Combining the results of Equations (10), (11), (13), (14), and (18), we obtain

(19) $$\prod =\frac{1}{2}\int_{-L_{c}}^{\infty }E_{f2}I_{f}(\frac{d^{2}\omega }{dy\prime^{2}}{)}^{2}dy\prime +\frac{1}{2}A_{m}G_{m}{\left(\frac{r_{m}}{r_{f}}\right)}^{2}\int_{-L_{c}}^{\infty }{\left(\frac{d^{2}\omega }{dy\prime^{2}}\right)}^{2}dy\prime -F_{u}\omega {|}_{y\prime =0}+\frac{E_{b}}{k}\int_{-L_{c}}^{\infty }\omega^{2}dy\prime .$$

According to the principle of minimum potential energy, the system is always stable in the state where its total potential energy is the smallest. From the variational method, the established condition of Equation (19) is

(20) $$E_{f2}I_{f}\omega^{\left(4\right)}-A_{m}G_{m}{\left(\frac{r_{m}}{r_{f}}\right)}^{2}\omega^{\prime \prime }+\frac{2E_{b}}{k}\omega =0.$$

Considering the semi-infinite beam hypothesis, its general solution is as follows [15,17]:

(21) $$\omega (y\prime )=e^{-\alpha *y\prime }(C_{1}\cos(\beta *y\prime )+C_{2}\sin(\beta *y\prime )),$$

where $\alpha *=\sqrt{\sqrt{\frac{Q*}{4E_{f2}I_{f}}}+\frac{N*}{4E_{f2}I_{f}}}$, $\beta *=\sqrt{\sqrt{\frac{Q*}{4E_{f2}I_{f}}}-\frac{N*}{4E_{f2}I_{f}}}$, $N*=A_{m}G_{m}{\left(\frac{r_{m}}{r_{f}}\right)}^{2}$,

(22) $$Q*=\frac{2E_{b}}{k}.$$

Equation (19) is the general form of the deflection equation. Qi et al. used the superposition principle to give the specific process of the solution of Equation (20) with *F_u_* as the boundary condition [21]. The solution of the Equation (20) can determine the relationship between *F_u_* and RVE deflection *ω* [15]. When the carbon fiber is deformed, the stress value is

(23) $$\sigma^{f}=E_{f2}\eta \omega \prime \prime ,$$

where *η* is the distance from each point on the fiber to the neutral axis. *η* can be positive or negative (−*r_f_* ≤ *η* ≤ *r_f_*) for tensile or compressive stresses. The breaking condition, depending on the maximum stress criterium (see Section 1) is at the maximum value; thus, when the fiber breaks,

(24) $$E_{f2}r_{f}\omega \prime \prime =X_{T}.$$

The above formula determines the relationship between the deflection and the ultimate strength of the fiber. Finally, it is possible to obtain the expression of the normal concentrated force *F_u_*_,_ The definition of the deflection based on the energy and the maximum stress at the break condition allows defining the force *F*u, following the description in [15].

(25) $$F_{u}=\frac{4\alpha *\beta *I_{f}X_{T}e^{2\alpha *L_{c}}}{r_{f}[\beta *e^{2\alpha *L_{c}}-\beta *\cos(2\beta *L_{c})+\alpha *\sin(2\beta *L_{c})]}.$$

Considering the effect of friction, the cutting force at the first breaking point in zone II (*F*_2.1_) can be obtained from the following geometric relationships [15,17]:

(26) $$F_{2.1}{{}_{\_}}_{x}=F_{u}\sin\theta +\mu F_{u}\cos\theta ,\;F_{F_{2.1}{}_{\_}y}=-F_{u}\cos\theta +\mu F_{u}\sin\theta ,$$

where *μ* is the tool–fiber friction coefficient.

Step 2: Calculation method of the cutting force at the residual fiber

As described in the results of finite element simulations in Section 3, for a single fiber, after the initial break at point P, the residual fibers still interact with the tool teeth, and the fibers on the upper part of point P become chips and are discharged along the rake face. The fibers below the point P (residual fibers) come into contact with the tool between point P and the lowest point B as the cutter teeth move further, and the residual fibers are further destroyed under the squeeze of the teeth.

**Figure 11:**
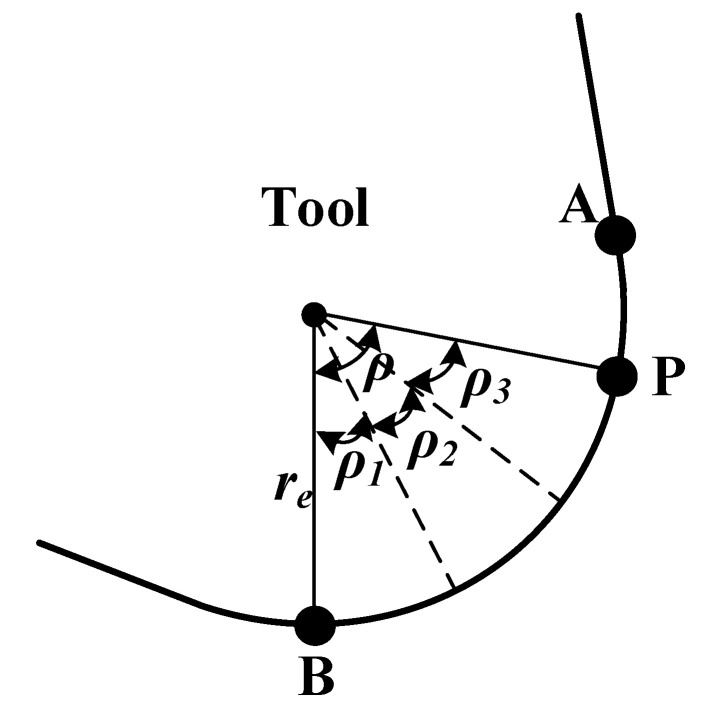
The schematic diagram of cutter tooth segmentation.

Residual fibers may break multiple times during the cutting process, which is difficult to describe with a continuous model. However, the removal process of residual fibers can be simply summarized as follows: the residual fibers discontinuously “sweep” from point P of the cutter to the lowest point B of the cutter. The fundamental reason for the cutting force generated in this process is that the cutter teeth squeezed the residual fiber; therefore, this paper simplified this process to the process of multi-segment cutter teeth extruding the composite material. As shown in Figure 11, the arc PB on the tooth is divided into n segments according to the angle *ρ*, where

(27) $$\rho_{1}=\rho_{2}=\cdot \cdot \cdot =\rho_{n}=\Delta \rho ,$$

(28) $$\rho_{n}\leq \Delta \rho ,$$

where Δρ is the angle range selected. According to the geometric relationship, we can get *ρ = θ.* For this work, we set Δρ = 5°. Since Δρ is difficult to measure experimentally, the values selected for Δρ were derived from the analysis of the FE results.

**Figure 12:**
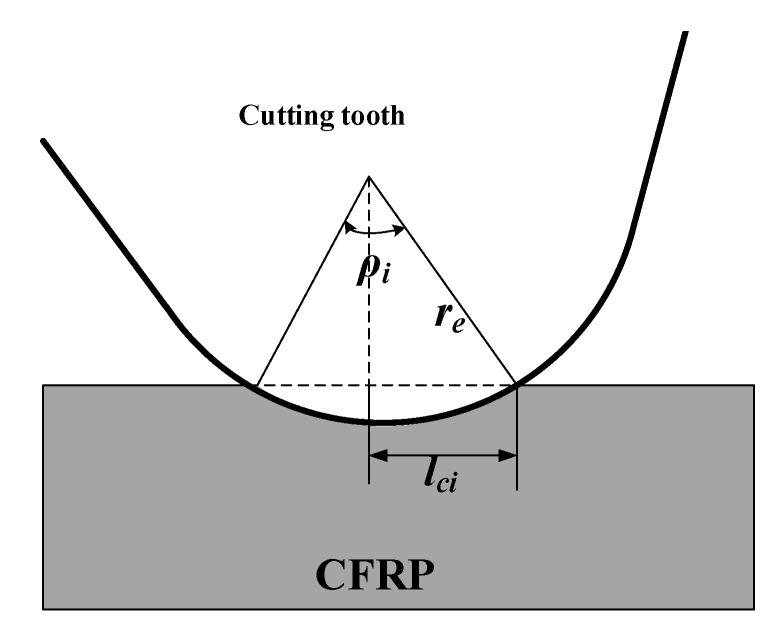
The schematic diagram of single-stage compression of cutter teeth.

The interaction between each segment of the cutter tooth and the residual fiber is independent, which is equivalent to the process of a cylinder extruding the elastic body, as shown in Figure 12. The force of CFRP on each tooth segment is calculated as follows [14,17]:

(29) $$F_{i}=\frac{L_{c}^{2}{}_{i}\times \pi \times E_{b}\times b}{4r_{e}}.$$

It can be known from the following geometric relationship:

(30) $$L_{c}{}_{i}=r_{e}\sin\frac{\rho_{i}}{2}.$$

**Figure 13:**
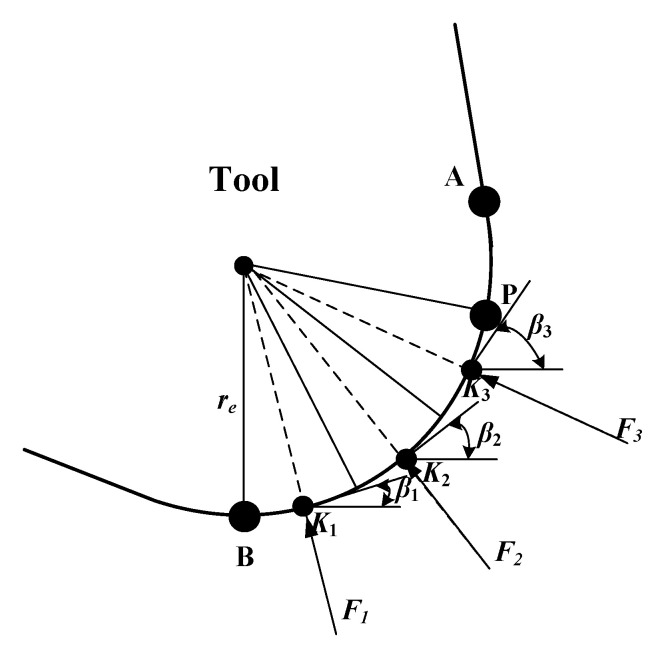
Drawing of the cutter tooth exerted forces per segment: the force per segment is located at the segment center (*K_i_*). The angle *β_i_* is plotted.

The schematic diagram of the positive pressure of the cutter tooth is shown in Figure 13. In order to facilitate the calculation, the force on each segment of the cutter tooth is treated as the concentrated force acting on the midpoint *K_i_*. The cutting force components per segment *F*_2.2*i*_ in the *x-* and *y*-directions are defined considering the effect of the CFRP friction force on the cutter tooth.

(31) $$F_{2.2i\_x}=F_{i}\sin(\beta_{i})+\mu F_{i}\cos(\beta_{i}),\;F_{2.2i\_y}=F_{i}\cos(\beta_{i})+\mu F_{i}\sin(\beta_{i}),$$

where *μ* is the tool–fiber friction coefficient, and the angle *β_i_* (see Figure 13) is defined according to

(32) $$\beta_{i}=\frac{\rho_{i}}{2}\sum_{j=1}^{i-1}\beta_{j}.$$

Considering that there will be a certain contact gap between the residual fiber and the cutter tooth during the removal process, the total cutting force generated by the cutter tooth PB segment is

(33) $$F_{2.2\_y}=k_{2.2}\sum_{i=1}^{n}F_{2.2i\_y},\;F_{2.2\_x}=k_{2.2}\sum_{i=1}^{n}F_{2.2i\_x},$$

where the factor *k*_2.2_ reflects the fact that there will be a certain contact gap between the residual fiber and the cutter tooth during the removal process (see Figure 7). This correction factor is mainly affected by the radius of the tool circle (*r_e_*). According to experience and experimental results, this paper concludes that the calculation formula of *k*_2.2_ is $k_{2.2}=\frac{7.5}{r_{e}}$.

### 4.3. Rebound Zone (Zone Ⅲ)

**Figure 14:**
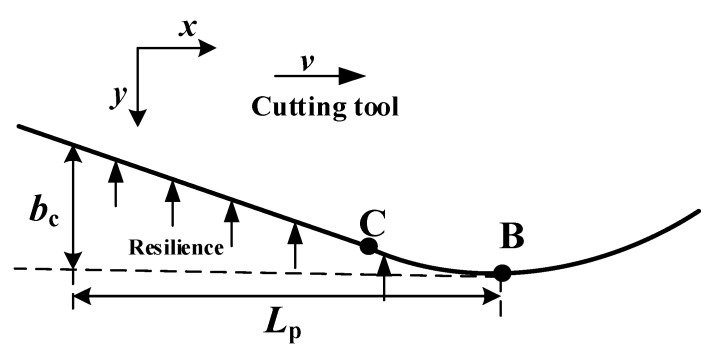
The schematic diagram of rebound zone.

In zone Ⅲ (see Figure 9 and Figure 14), the composite material under the cutting edge rebounds to the height of *b_c_*, and the distance between the rebound composite material and the tooth in the direction of the tooth movement is *L_p_*_._ The CB arc is regarded as an oblique straight line with an inclination angle of α (the clearance tool angle, see Section 5). The force can be regarded as a two-dimensional wedge squeezed on an elastic surface. The calculation method of *L_p_* can be obtained [17] according to the geometric relationship in Equation (34).

(34) $$L_{p}=\frac{b_{c}}{\tan(\alpha )}.$$

According to the contact mechanics, the flank surface extends beyond the contact boundary, the pressure on the boundary must be reduced to zero, and the pressure on the flank surface is as follows [17]:

(35) $$F_{p}=\frac{b\cdot L_{p}\cdot E^{*}\cdot \tan(\alpha )}{2}=\frac{b\cdot b_{c}\cdot E^{*}}{2},$$

where *b* is the thickness about the CFRP laminate, and $E^{*}$ is the equivalent elastic modulus of the material after cutting. The value of $E^{*}$ should be less than the elastic modulus before CFRP processing. For the convenience of calculation, the authors of [14,15] assumed that the rebound height of the material is equal to *r_e_*, $E^{*}=E_{m}$. The *r_e_* parameter used in this analytical model is not a fixed value and can take different values. The effect of *r_e_* on the cutting force is studied in Section 5. The rebound height of the processed material is a function related to *r_e_*, and this paper adjusted it according to experimental results and experience as $b_{c}=r_{e}(1-0.3\frac{r_{e}-15}{35})$.

Similarly, considering the friction in the cutting process in this zone, the cutting force *F*_3_ can be obtained as follows:

(36) $$F_{3\_x}=F_{p}{\left(\cos(\alpha )\right)}^{2},\;F_{3\_y}=F_{p}(1+\mu \cdot \cos(\alpha )\cdot \sin(\alpha )),$$

where μ is friction coefficient between the tool and fiber.

### 4.4. Cutting Force Evaluation

In summary, the total cutting force of CFRP can be obtained as follows:

(37) $$F_{x}=F_{1\_x}+F_{2.1\_x}+F_{2.2\_x}+F_{3\_x},\;F_{y}=F_{1\_y}+F_{2.1\_y}+F_{2.2\_y}+F_{3\_y}.$$

The calculation of $F_{2.2\_x}$ and $F_{2.2\_y}$ comes from the finite element analysis results. $F_{2.2\_x}$ and $F_{2.2\_y}$ depend on the values of parameters Δρ and *k*_2.2_, which are evaluated according to the FE model.

## 5. CFRP Cutting Force Analytical Model Verification

### 5.1. Experimental Design

In order to verify the accuracy of the analytical model of CFRP cutting force theory proposed in this paper, a CFRP side milling experiment was designed. A T700 unidirectional laminate with a fiber volume fraction of 60% was selected for the experiment, and the diameter of a single-fiber filament was 7 μm. The thickness of each layer of carbon fiber was 0.2 mm, and there were 20 layers in total, making a 4 mm thick plate. The size was about 65 × 100 mm^2^. The material parameters of each component of CFRP are shown in Table 1, Table 2 and Table 3. According to the findings of Yan et al., the Johnson–Cook parameters of epoxy resin are A = 27 MPa, B = 654.18 Mpa, n = 0.772, c = 0.124, and m = 0.304 [21,22]. The Johnson-Cook parameters allow properly describing the plastic range, as described in Figure 4.

**Figure 15:**
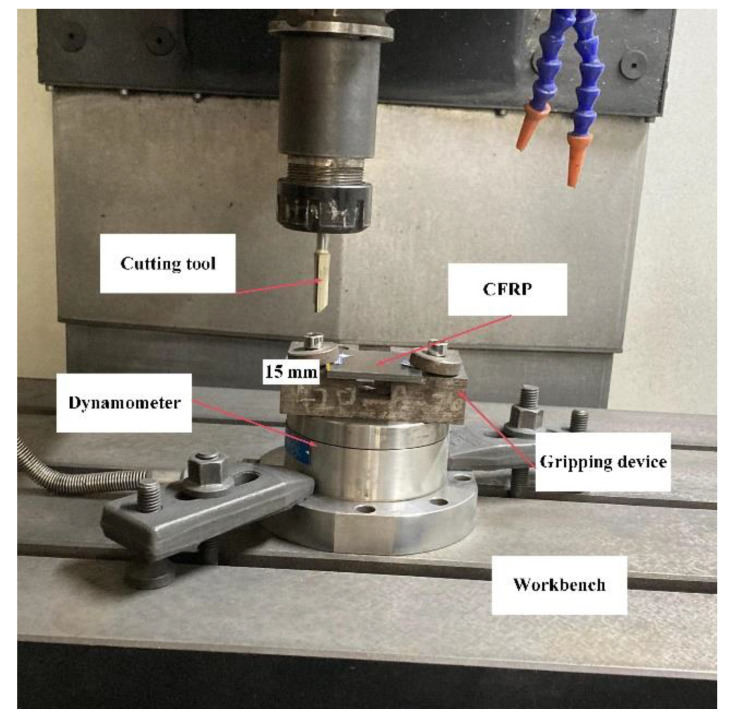
Experimental device layout.

The cutting tool was a customized double-edged straight milling cutter. The material of the cutting tool was uncoated cemented carbide. The material parameters of the cutting tool are shown in Table 4. The diameter D of the milling cutter was 10 mm, the tool edge radius was 15 μm, the rake angle was 10°, and the clearance angle α was 15°. The machine tool used in the milling process was an XK714D vertical machining center produced by Hanchuan Machinery Factory (Hanzhong, China) equipped with a Siemens 828D system (Siemens Aktiengesellschaft, Munich, Germany). During the experiment, Kistler9272 (Kistler Instrumente AG, Winterthur, Switzerland) was used to measure the milling force, and the force data collection frequency was 2000 Hz. In the experiment, the experimental repetition error of the cutting force measurement was less than 3%. The experiment layout is shown in Figure 15. In the experiment, the CFRP was fixed by bolts, and the distance between the fixing bolts and the machined surface was about 15 mm. As can be seen, the CFRP was clamped in a simple manner, but the clamping effect was equivalent, using this approach, to the bottom/side fixed surface set in the FE model. The experimental parameters are shown in Table 5.

### 5.2. Cutting Force Model Verification

**Figure 16:**
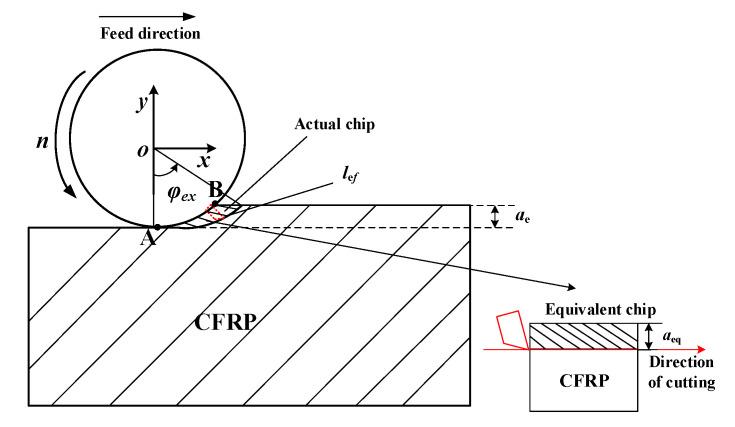
The schematic illustration of single-tooth cutting.

The milling cutter used in the experiment in this chapter was a straight-edge milling cutter. During the machining process, the instantaneous cutting thickness changed continuously. In the experiment, the radial depth of cut *a_e_* was small. In order to complete the verification of the model, the milling process was simplified to a two-dimensional right-angle cutting process [16]. Figure 16 shows a schematic illustration of single-tooth cutting. The cutter teeth cut into the material at point A and cut out of the material at point B. The rotation angles of the cutter at point A and point B were 0° and *φ_ex_*, respectively. Since the cutting speed (261.7 mm/s) is much higher than the feed speed (3.8 mm/s) during the milling process, when the milling cutter makes one revolution the effective cutting distance of each tooth is

(38) $$l_{ef}=\frac{D\varphi_{ex}}{2},$$

where $\varphi_{ex}$ can be calculated by the following geometric relation:

(39) $$\varphi_{ex}=\arccos(1-\frac{2a_{e}}{D}).$$

When the milling cutter makes one revolution, the zone of the material removed by each tooth is

(40) $$A_{ef}=v_{f}a_{e}.$$

Thus, the equivalent depth of cut is

(41) $$a_{eq}=\frac{A_{ef}}{l_{ef}}=\frac{2\nu_{f}a_{e}}{D\varphi_{\mathrm{ex}}}.$$

The force was recorded throughout the cutting process. The data were sorted to define a stable cutting range to define the measured average force $\bar{F}$. Milling is an intermittent cutting process. The average cutting force ${\bar{F}}_{ef}$ in the actual cutting process can be obtained by the following formula, relating the continuous measured force to the actual cutting teeth:

(42) $${\bar{F}}_{ef}=\frac{m\varphi_{ex}\bar{F}}{2\pi },$$

where *m* is the number of teeth.

**Figure 17:**
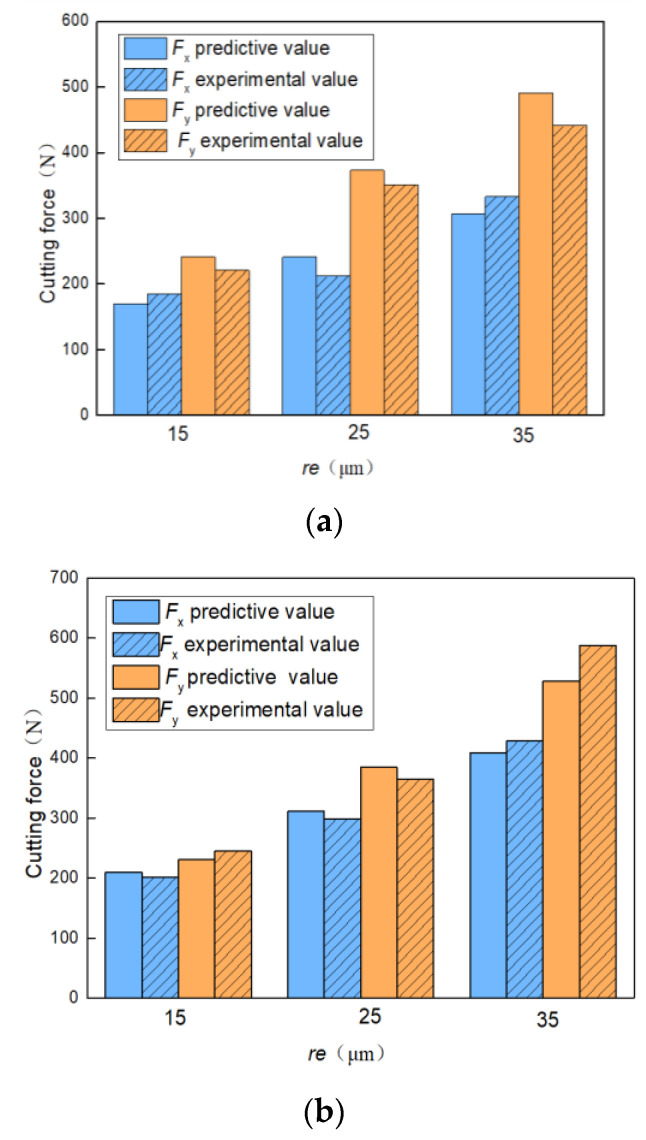
Comparison of cutting force: measured values and model prediction values for fiber cutting angles (**a**) *θ* = 45° and (**b**) *θ* = 90°, as a function of the tool edge radius (*r_e_*).

The experimental value of the measured cutting force and the predicted value of the model are shown in Figure 17, according to Equations (37) and (42), respectively. The forces are shown for fiber cutting angles 45° (a) and 90° (b), as a function of the tool edge radius (*r_e_*). The comparison shows that the model directly reproduced the measured values for the two cutting angles, for both *x-* and *y*-components, and for the tool edge radius dependence. The results show clearly that the cutting force increased directly with increasing values of the tool edge radius, being a key parameter to define the cutting force value. The maximum relative deviation found was below 15%, indicating that the theoretical model for the CFRP cutting force considering the tool edge circle has a sound capability to provide force values within a limited deviation range.

## 6. Conclusions

This paper studied the mechanism of CFRP material removal during cutting on the basis of the CFRP finite element cutting model, and established an analytical model of CFRP cutting force considering the radius of the edge circle. The main conclusions are as follows:

1. In the CFRP cutting process, the tool tooth and a single carbon fiber first come into contact, eventually breaking at that point. The residual fibers may also interact with the tool teeth and break multiple times.
2. The tool edge radius has a direct influence on the cutting force, and the model correctly reflects the dependence in the different cases analyzed on the fiber cutting angle.
3. The comparison with measured forces shows that the maximum relative deviation of the model predicted value of the cutting force was below 15%, which proves that the analytical model built for the CFRP cutting force can provide values within a limited uncertainty, useful for many evaluations and machining applications.

The theoretical analysis model of the CFRP cutting process has been continuously completed and improved through different academic proposals including different details and dependences. The model proposed in this paper considers the multiple breakage of the single fiber and adds the influence of the edge radius on the cutting process.

## Figures and Tables

**Table 1:** Carbon fiber material parameters [21,22].

Density	Longitudinal Elastic Modulus	Transverse Elastic Modulus	Poisson’s Ratio	Shear Modulus		Tensile Strength		Compressive Strength	
ρ (kg/m^3^)	*E_f_*_1_ (GPa)	*E_f_*_2_ (GPa)		*G*_12_ = *G*_13_ (GPa)	*G*_23_ (GPa)	X_t_ (GPa)	Xc (GPa)	Y_t_ (GPa)	Y_c_ (GPa)
1760	230	8.2	0.2	28	5.5	4.9	3.96	3.34	1.5

**Table 2:** Performance mechanical parameters of epoxy resin [19,21].

Elastic Modulus	Poisson’s Ratio	Shear Modulus	Shear Strength	Density	Yield Strength	Damage Plastic Strain	Fracture Energy
*E_m_* (GPa)		*G_m_* (GPa)	$\tau_{c}\;(\mathrm{MPa})$	ρ (kg/m^3^)	$\tau_{c}\;(\mathrm{MPa})$	${\bar{\varepsilon }}_{0}^{pl}$	$G_{C}^{m}\;(N/\mathrm{mm})$
3.5	0.33	1.02	62	980	27	5%	0.1

**Table 3:** The interface material parameters [22,23].

Normal Strength	Shear Strength	Elastic Stiffness	Fracture Energy		Mixed-Mode
$t_{n}^{0}$ (MPa)	$t_{s}^{0}$ $=t_{t}^{0}\;(\mathrm{MPa})$	*K_nn_ = K_ss_ = K_tt_* (N/mm^3^)	$G_{1}^{C}\;(N/\mathrm{mm})$	$G_{2}^{C}=G_{3}^{C}\;(N/\mathrm{mm})$	*η_a_*
167.5	25	100,000	0.002	0.006	1.45

**Table 4:** Tool material parameters [21].

Density (kg/m^3^)	Elastic Modulus (GPa)	Poisson’s Ratio
14,450	640	0.22

**Table 5:** The machining parameters used for the tests are shown. The cutting angles were 45° and 90°. The edge radii of the tools were 15, 25, and 35 μm.

Spindle Speed	Cutting Speed	Fiber Cutting Angle	Radial Depth of Cut	Axial Depth of Cut	Feed per Tooth	The Cutter Radius
*n* (r/min)	*v* (mm/s)	*θ* (°)	*a_e_* (mm)	*a_p_* (mm)	*v_f_* (mm/tooth)	*r_e_* (μm)
500	3.8	45°/90°	1	4	0.225	15/25/35

## Data Availability

Not applicable.

## Abbreviations

Some important parameters in this paper are defined below.

*E_m_*	Elastic modulus of the matrix	*r_e_*	Radius of the edge circle
*θ*	Fiber cutting angle	*t_n_*	Normal stress
*E_b_*	Equivalent Young’s modulus	*t_s_, t_t_*	Shear stresses
*γ*	Rake angle of the tool	*a_c_*	Cutting depth
$U_{f}$	Fiber strain energy	*b*	Thickness about CFRP laminate
*E_f_*_1_	Longitudinal elastic modulus	*G*_12_	Shear modulus
$I_{f}$	Moment of inertia of the section	*r_e_*	Radius of the edge circle
$U_{m}$	Matrix strain energy	*k*	Dimensionless number
*G*_m_	Shear modulus of the matrix	$r_{m}$	Radius of the RVE
$g_{C}^{m}$	Fracture energy density	$r_{f}$	Radius of the carbon fiber
$G_{C}^{m}$	Fracture energy	$V_{f}$	Fiber volume fraction
*U*_m_	Matrix strain energy	${\bar{F}}_{ef}$	Average cutting force
$W_{F_{u}}$	Work done by the the external force	$U_{f}$	Fiber strain energy
$W_{P_{b}}$	Work done by the supporting force	$F_{p}$	Pressure on the flank surface
$E_{f}{}_{2}$	Transverse elastic modulus of fiber	*a_e_*	Radial depth of cut
